# Integrated Transcriptomic and Proteomic Analysis of the Stress Response Mechanisms of *Micractinium* from the Tibetan Plateau Under Leather Wastewater Exposure

**DOI:** 10.3390/biology15020123

**Published:** 2026-01-09

**Authors:** Haoyu Wang, Bo Fang, Geng Xu, Kejie Li, Fangjing Xiao, Qiangying Zhang, Duo Bu, Xiaomei Cui

**Affiliations:** Key Laboratory of Biodiversity and Environment on the Qinghai-Tibet Plateau, Ministry of Education, School of Ecology and Environment, Xizang University, Lhasa 850001, China; wanghaoyu1279@163.com (H.W.); fangb1994@gmail.com (B.F.); xgydsa@163.com (G.X.); 23210307035@stu.utibet.edu.cn (K.L.); xfj2023@stu.utibet.edu.cn (F.X.); zhangqiangying@utibet.edu.cn (Q.Z.)

**Keywords:** Plateau *Micractinium* sp., ammonia nitrogen removal, tannery wastewater, omics technology

## Abstract

In this study, a strain of green microalga adapted to the extreme environmental conditions of the Tibetan Plateau was isolated from the Lalu Wetland. The isolate was identified and tentatively designated as *Micractinium* sp. LL-1. Following the inoculation of strain LL-1 into tannery wastewater, the ammonia nitrogen concentration was rapidly reduced, achieving a removal efficiency of 98.7%. The maximum accumulated biomass reached 1641.68 mg/L and 1461.28 mg/L. Integrated transcriptomic and label-free quantitative proteomic approaches were employed to systematically investigate the molecular response mechanisms of LL-1 under tannery wastewater stress. Transcriptomic analysis revealed that differentially expressed genes were enriched in pathways related to cell proliferation, morphogenesis, intracellular transport, protein synthesis, photosynthesis, and redox processes. Proteomic analysis indicated that LL-1 enhances cellular and enzymatic activities, strengthens regulatory capacity, modulates key metabolic pathways, and upregulates stress-responsive proteins. Under tannery wastewater stress, LL-1 exhibits dynamic adaptation involving signal perception and metabolic reconfiguration through the coordinated regulation of multiple pathways. Specifically, ribosomal translation and nucleic acid binding regulate biosynthetic capacity; the redistribution of energy metabolism boosts photosynthetic carbon fixation and ATP generation; and membrane transport coupled with antioxidant mechanisms mitigates stress-induced damage. Collectively, this study provides theoretical insights into microalgal adaptation to complex wastewater environments and offers potential targets for strain improvement and wastewater valorization.

## Simple Summary

Microalgae demonstrate strong environmental resilience and high pollutant removal efficiency in tannery wastewater treatment. In this study, *Micractinium* sp. strain LL-1 was isolated from the Lalu Wetland on the Tibetan Plateau. Transcriptomic and proteomic analyses were integrated to elucidate its molecular response mechanisms to tannery wastewater stress. Results revealed that strain LL-1 exhibits robust adaptability by regulating key biological processes, including cell proliferation, morphological development, protein synthesis, and photosynthesis. Specifically, transcriptomics highlighted significant alterations in the cytoskeletal microtubule system and photosynthetic pathways, while proteomics showed that the strain enhances tolerance via the modulation of energy metabolism, antioxidant mechanisms, and pollutant efflux. These findings provide novel theoretical insights into microalgal-based wastewater treatment and offer potential strategies for resource recovery and environmental remediation.

## 1. Introduction

The Tibetan Plateau is the primary region for yak husbandry in China, and the rising living standards of local residents, together with the rapid growth in plateau tourism, have driven a substantial increase in the demand for leather products. Yak hides, a by-product of the pastoral economy, serve as an essential raw material for the regional leather industry [1]. However, processing a single hide requires approximately 0.3–1.0 tons of water, and large-scale leather production generates substantial volumes of wastewater [2]. If discharged without adequate treatment, this wastewater poses serious risks to the plateau’s already fragile and highly sensitive ecosystem [3]. Leather processing typically involves the use of hundreds of chemicals, including sulfides, chlorides, surfactants, organic solvents, and ammonia-based compounds [4], while various processing steps release organic nitrogen, non-collagen proteins, and other pollutants into the effluent [5,6]. Consequently, leather wastewater is characterized by high salinity, elevated ammonia nitrogen, intense coloration, and the presence of heavy metals. These characteristics render its treatment substantially more challenging than that of conventional industrial wastewater. In particular, insufficient removal of ammonia nitrogen can lead to eutrophication [7], oxygen depletion [8], and the degradation of habitats for algae and aquatic organisms [9]. Therefore, promoting sustainable transition within the leather industry and safeguarding ecological security have become urgent and critical priorities.

Currently, the main technologies for treating leather wastewater include air stripping [10] and ion exchange [11], both of which are well-established, readily controllable, and relatively simple to operate. However, physical processes are costly for ammonia nitrogen removal and may cause scaling and release ammonia gas under alkaline conditions, leading to secondary pollution. In contrast, chemical methods possess limited adsorption capacity and are effective only at low to moderate ammonia nitrogen concentrations. These limitations render both methods poorly suited for high-altitude regions, where low oxygen levels, reduced atmospheric pressure, fragile ecosystems, and geographic remoteness pose additional challenges. In recent years, microalgae-based biological methods for ammonia nitrogen removal have shown considerable potential [12,13]. Compared with physical and chemical treatments, microalgal bioremediation reduces reagent consumption, minimizes secondary pollution [14], and generates recoverable algal biomass, thereby enhancing the sustainability of resource utilization [15]. Wang et al. [16] reported that *Chlorella* sp. JSC-6 achieved a 91.3% removal efficiency for ammonia nitrogen from swine wastewater under mixotrophic cultivation conditions. Pang et al. [17] isolated a highly ammonia-tolerant strain, *Chlorella* sp. CA1, from dairy wastewater, which exhibited an ammonia tolerance more than 20 times greater than that of conventional Chlorella strains. Similarly, Li et al. [18] compared the pollutant removal performance of two Chlorella strains in municipal wastewater and found that the maximum ammonia nitrogen removal rates reached 80.5% and 66.8%, respectively, while total phosphorus removal rates were as high as 87.4% and 86.1%. However, the physiological and molecular mechanisms by which microalgae remove ammonia nitrogen remain poorly understood and require further elucidation.

The Tibetan Plateau, characterized by extensive lakes, rivers, and wetlands, constitutes an extreme environment marked by cold temperatures, low oxygen, intense radiation, and limited precipitation [19]. These conditions give rise to unique ecological niches that support diverse microalgal communities, which may harbor novel adaptive genetic resources capable of revealing the physiological and molecular mechanisms of ammonia nitrogen removal [20]. Recent advances in omics technologies have substantially improved our understanding of microalgal environmental adaptation and expanded the application of microalgae in bioenergy production and pollution control [21]. Transcriptomics systematically reveals changes in gene expression and the regulation of metabolic pathways under specific stress conditions [22,23], whereas proteomics elucidates relationships between protein function and metabolic activity at both translational and post-translational levels [24]. The integration of omics technologies enables more precise identification of stress-responsive genes, key enzymes, and stress-tolerant proteins involved in ammonia nitrogen removal. These insights provide valuable targets for future genetic engineering and adaptive evolution aimed at enhancing ammonia nitrogen removal efficiency.

In this study, a dominant, high-biomass algal strain was isolated from the Lalu Wetland on the Tibetan Plateau. Integrated transcriptomic and proteomic analyses were conducted to characterize differential gene and protein expression profiles, as well as enriched metabolic pathways, under tannery wastewater stress, thereby elucidating the molecular mechanisms underlying the strain’s response to pollutants. This work not only provides a novel biological resource for investigating the taxonomy and adaptive mechanisms of microalgae inhabiting extreme plateau environments but also offers theoretical insights to guide the development of microalgae-based strategies for wastewater bioremediation and resource recovery.

## 2. Materials and Methods

### 2.1. Collection of Microalgae

Water samples were collected from the Lalu Wetland on the Tibetan Plateau (29°40′34″ N, 91°06′09″ E). A 30 µm plankton net was towed in a figure-eight pattern at a speed of 20–30 cm/s from the surface to a depth of 0.5 m for 1–3 min. Approximately 100 L of water was filtered, and the concentrates were collected in a 50 L polytetrafluoroethylene container. Under aseptic conditions, the mixed water sample was filtered through a 0.45 μm membrane. The retained material was inoculated into liquid medium and pre-cultured in an illuminated incubator at 25 ± 1 °C under a 12 h:12 h light-dark cycle with a light intensity of 37 µmol photons m^−2^ s^−1^ and three parallel replicates. The cultured suspension was serially diluted and streaked onto solid medium. Cultures were incubated under illumination for 7–14 days until distinct colonies appeared. Individual colonies exhibiting high biomass were selected and examined microscopically. Colonies confirmed as single algal species were considered successfully isolated and purified [25]. The isolated microalga was identified as *Micractinium* sp. (GenBank accession number: PRJNA81323). Detailed identification results are provided in Table S2 and Figure S1. The isolated strain was tentatively designated as *Micractinium* sp. LL-1.

### 2.2. Collection of Tannery Wastewater and Microalgae Cultivation

Tannery wastewater was collected from the effluent outlet of a tannery in Lhasa**. The ** collected samples were stored in PTFE containers and kept in a refrigerated vehicle at 4 °C during transportation, ensuring delivery to the laboratory within 2 h for further analysis. All filtration equipment and containers were sterilized at 121 °C under 1.013 MPa pressure**. The ** wastewater was filtered through a 0.22 µm sterile membrane filter to obtain sterile wastewater. The physicochemical properties of the wastewater are summarized in Table S1. BG11 medium was prepared according to the standard formulation described in Supplementary Material Table S3. An appropriate volume of algal culture in the logarithmic growth phase was centrifuged at 12,000 r/min for 10 min. The resulting algal pellet was carefully collected, washed three times with sterile ultrapure water, and gently resuspended to minimize shear-induced cell damage. Cell integrity and the absence of contamination were verified under an optical microscope to ensure viability and atelicity.

Six independent 1 L microalgal cultures were prepared. Before the experiment, all pre-cultures were grown to the logarithmic phase. The optical density (OD_682_) of each algal sample was measured to confirm consistent initial biomass across groups. Three replicates were randomly assigned 1 L of pre-treated tannery wastewater and designated as the wastewater experimental group (LL_2), with sample names LL_2_1, LL_2_2, and LL_2_3. The remaining three replicates received 1 L of BG11 liquid medium each and served as the medium control group (LL_3), with sample names LL_3_1, LL_3_2, and LL_3_3. All six cultures were manually agitated three times daily at fixed time points to ensure uniform dispersion of the algal biomass and prevent settling. To avoid contamination, the culture vessels were sealed with parafilm and incubated in a light chamber. The groups were cultivated continuously for 26 days. OD_682_ was measured every two days at the same time point to monitor the growth of LL-1, and ammonia nitrogen concentration was determined to evaluate removal efficiency. All measurements were performed in triplicate. At the end of the cultivation period, algal pellets were collected by centrifugation, flash-frozen in liquid nitrogen, and stored for subsequent RNA and protein extraction.

### 2.3. Dry Weight (DW) and Optical Density (OD) of Micractinium sp.

A UV-visible spectrophotometer was used to perform full-wavelength spectral scans on the LL-1 algal culture, BG11 blank, and wastewater blank, respectively, to identify the characteristic absorption peak of LL-1 and thereby determine the optimal wavelength (OD_682_). A 0.45 µm filter membrane was dried at 60 °C to constant weight, with the mass difference between two consecutive weightings being less than 0.2 mg. The mass of the blank membrane was recorded as m_0_. A known volume V of algal culture was then filtered, and the membrane was dried again at 60 °C to constant weight m_1_, biomass concentration was calculated using the following Formula (1). Algal cultures of varying concentrations were used to measure OD_682_ values, with corresponding DW determinations to generate multiple datasets. A linear regression equation was established (R^2^ > 0.99). All subsequent OD measurements were diluted to within the linear range of the regression and back-calculated to the original sample using the dilution factor. In later experiments, DW and biomass concentration of LL-1 were calculated directly from optical density measurements.

(1) $$DW=\frac{M_{1}-M_{0}}{V}\times 1000$$

M_0_ is the mass of the filter membrane before filtration (mg), m_1_ is the constant weight mass after filtration (mg), V is the filtration volume (mL).

### 2.4. Detection Indicators of Microalgae Growth and Ammonia Nitrogen Removal

The concentration of ammonia nitrogen in water samples was determined strictly following the national standard method (HJ 535-2009) using Nessler’s reagent spectrophotometry [26]. Briefly, 10 mL of filtered sample was transferred to a 50 mL colorimetric tube and diluted to the mark with distilled water. Subsequently, 1 mL of sodium potassium tartrate solution and 1.5 mL of Nessler’s reagent were added in sequence. After incubating for 10 min to allow full color development, the absorbance was measured at 420 nm using a UV-visible spectrophotometer. For each batch of measurements, a standard curve was constructed using NH_4_^+^-N standard solutions at least 5–7 concentration points, with a required coefficient of determination (R^2^) ≥ 0.99. Samples exceeding the linear range were appropriately diluted prior to analysis, and the results were back-calculated to the original concentration. The ammonia nitrogen removal efficiency and average consumption rate by LL-1 were calculated using Equations (2) and (3), respectively, while the specific growth rate (µ, %) and biomass productivity of LL-1 (P, mg/L/d) were determined using Equations (4) and (5).

(2) $$\eta =\frac{C_{0}-C_{a}}{C_{0}}\times 100\%$$

(3) $$∆C=\frac{C_{0}-C_{a}}{T_{i}-T_{0}}\times 100\%$$

In the formula, C_0_ and C_a_ (mg/L) represent the ammonia nitrogen concentrations at the initial time of the experiment T_0_ and at the end of the experiment T_a_, respectively, while C_i_ denotes the ammonia nitrogen concentration at any arbitrary time point T_i_.

(4) $$\mu =\frac{\ln N_{2}-\ln N_{1}}{T_{2}-T_{1}}$$

(5) $$P=\frac{N_{2}-N_{1}}{T_{2}-T_{1}}$$

T represents the cultivation time (in days), while N_2_ and N_1_ denote the biomass concentrations (mg/L) of the microalga on day T_2_ and day T_1_.

### 2.5. Transcriptomic and Proteomics Analysis

Transcriptomic Analysis: Total RNA was extracted using a Plant Polysaccharide & Polyphenol RNA Extraction Kit (TIANGEN, Beijing, China). Genomic DNA was removed by digestion with RNase-free DNase I. RNA quality and quantity were assessed using an Agilent 2100 Bioanalyzer equipped with the RNA Nano 6000 Assay Kit (Agilent Technologies, Santa Clara, CA, USA). Three biological replicates were utilized for library construction. Sequencing was performed on an Illumina NovaSeq 6000 platform (Illumina, San Diego, CA, USA). De novo assembly of raw reads was conducted using Trinity [27], followed by hierarchical clustering via Corset [28]. Functional annotation and differential expression analyses were performed against the GO, KOG, and KEGG databases. Metabolic pathway enrichment analysis was conducted following previously described methods [29].

Proteomic Analysis: Label-free quantitative proteomics was performed to profile protein expression. Total protein was extracted using SDT lysis buffer. Protein concentration was determined using a Bradford Protein Assay Kit (Bio-Rad Laboratories, Hercules, CA, USA). After trypsin digestion, peptides were analyzed via LC-MS/MS on a Q Exactive™ HF-X mass spectrometer (Thermo Fisher Scientific, Waltham, MA, USA). Raw data were processed, and quantitative analysis was performed using Proteome Discoverer software (version 2.4, Thermo Fisher Scientific, Waltham, MA, USA). Differentially expressed proteins (DEPs) were identified using Student’s *t*-test, followed by functional annotation (GO and InterPro) and pathway enrichment analysis.

## 3. Results

### 3.1. Monitoring of the Growth of Micractinium sp. LL-1

**Figure 1:**
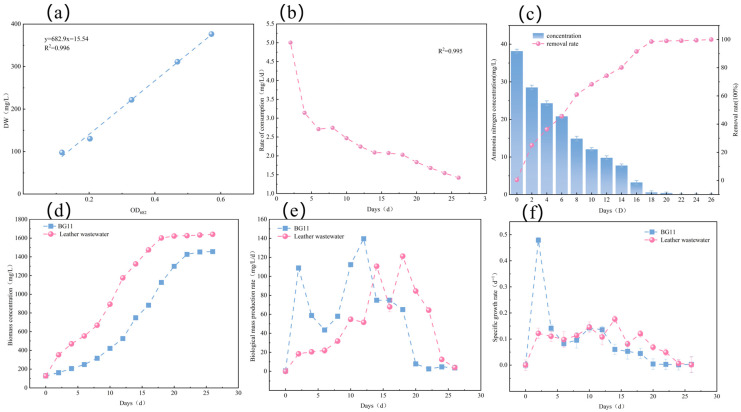
LL-1 growth status and ammonia nitrogen removal determination. (**a**) Relationship between OD_682_ and dry weight of LL-1. (**b**) Ammonia nitrogen consumption rate. (**c**) Changes in ammonia concentration and removal rate over time. (**d**) Growth curve of LL-1. (**e**) Biomass yield of LL-1. (**f**) Specific growth rate of LL-1.

To validate the biomass quantification method, the correlation between optical density (OD_682_) and dry cell weight (DW) was analyzed. As shown in Figure 1a, a strong linear correlation (R^2^ = 0.996) was observed for *Micractinium* sp. LL-1, confirming the reliability of optical measurements for biomass monitoring. Subsequently, a comparison of the growth curves (Figure 1d) and kinetic parameters (Figure 1f) between standard BG11 medium and tannery wastewater revealed distinct growth patterns. These differences reflect the physiological adjustments and stress response mechanisms of the microalgae when exposed to complex wastewater environments.

In control BG11 medium, LL-1 exhibited a negligible lag phase, immediately entering the exponential growth phase. A sharp, linear increase in biomass was observed from day 8 to day 18, corresponding to the peak of metabolic activity. Subsequently (after day 18), growth transitioned into the stationary and decline phases, attributed to nutrient depletion and the accumulation of metabolic byproducts. The maximum biomass reached 1641.7 mg/L. Notably, the specific growth rate peaked on day 2 before gradually declining. Overall, LL-1 achieved an average specific growth rate of 0.14 d^−1^ and an average biomass productivity of 81.6 mg/L/d (Figure 1e).

In contrast, LL-1 exhibited a distinct lag phase of 8 days when cultivated in tannery wastewater. This prolonged acclimation period is likely attributed to the inhibitory effects of high organic loads, heavy metals, or light limitation induced by the intense coloration of the wastewater. Such conditions likely necessitated metabolic reconfiguration and the synthesis of stress-responsive proteins. Following this acclimation phase, LL-1 demonstrated strong adaptability. From day 8, the strain established tolerance mechanisms, entered the exponential growth phase, and maintained stable biomass accumulation. The growth curve plateaued by day 22, reaching a maximum biomass of 1461.3 mg/L. The specific growth rate remained relatively stable (approx. 0.12 d^−1^), with an average of 0.11 d^−1^ and a biomass productivity of 51.1 mg/L/d. While these values were slightly lower than those observed in the BG11 control, the substantial biomass production in undiluted wastewater indicates the capability of LL-1 to utilize wastewater-derived carbon, nitrogen, and phosphorus for mixotrophic or heterotrophic growth.

### 3.2. Efficiency of Micractinium sp. LL-1 in Removing Ammonia Nitrogen

Figure 1c clearly illustrates the dynamics of ammonia nitrogen removal by LL-1 in the tannery wastewater culture system. Overall, the ammonia nitrogen concentration showed a marked and continuous decline over the cultivation period, while the removal efficiency exhibited a corresponding sigmoidal increase. Experimental data revealed that, within the first two weeks after inoculation, ammonia nitrogen in the wastewater was efficiently utilized by the microalgae, resulting in a phase of rapid depletion. Notably, although the microalgae experienced an acclimation period with relatively slow growth during the initial 8 days, nitrogen uptake did not cease. This suggests that during the pre-division acclimation stage, active physiological and biochemical adjustments occurred within the algal cells, with substantial nitrogen being assimilated for the synthesis of macromolecules such as proteins and nucleic acids to prepare for cell proliferation. By day 16, the ammonia nitrogen removal efficiency had exceeded 90%, indicating the strain’s robust tolerance and utilization capacity for high ammonia concentrations. From day 16 to day 22, as the culture progressed into the mid-to-late phase, ammonia nitrogen concentrations continued to decrease. On day 18, the concentration had dropped to 0.48 mg/L. At this point, despite the near-depletion of available nitrogen, the microalgae retained the ability to uptake residual ammonia nitrogen. By day 22, measurements indicated that ammonia nitrogen was nearly exhausted, with the final removal efficiency approaching 100%.

These findings demonstrate that LL-1 is capable of both tolerating high ammonia loads and achieving polishing of low-concentration effluent. The ammonia consumption rate closely paralleled the microalgal growth curve (Figure 1b,d). Specifically, during the exponential phase (days 8–18), rapid biomass accumulation triggered peak metabolic nitrogen demand, driving a sharp decline in ammonia concentration. The high goodness of fit (specify R^2^ here if not mentioned before, e.g., R^2^ > 0.99) indicates that the kinetic model accurately characterizes the ammonia consumption kinetics of LL-1 in tannery wastewater. Furthermore, the robust fit, combined with the growth-dependent removal pattern, suggests that ammonia removal is mediated primarily by enzyme-catalyzed biochemical processes rather than simple physical adsorption.

### 3.3. Sample RNA and Protein Quality Detection and Data Quality Control

RNA sequencing quality was validated prior to analysis. All samples exhibited RNA integrity numbers (RIN) ≥ 7.4, with no detectable impurities (e.g., pigments or polysaccharides). High-throughput sequencing generated 21.9–24.5 million clean reads per sample, with clean read rates consistently exceeding 93.16% and Q30 percentages above 94.84% (Table S3, Figures S2 and S3). The error rates (<0.05%) and GC content were within expected ranges, indicating high-quality sequencing data. For the transcriptome assembly, de novo assembly yielded 67,774 transcripts and 31,090 unigenes (Table S4). The N50 lengths were 2934 bp for transcripts and 2586 bp for unigenes, with average lengths of 1930 bp and 1613 bp, respectively. Lengths ranged from 301 bp to 21,286 bp. These metrics indicate a robust assembly quality suitable for downstream annotation.

Proteomic data quality was assessed via SDS-PAGE and mass spectrometry. SDS-PAGE revealed sharp, high-intensity bands around 50 kDa, confirming protein integrity. Following filtration (FDR < 1%), a total of 19,093 peptides and 3106 proteins were identified. Peptide lengths largely fell within the 7–23 residue range, consistent with standard tryptic digestion (Figure S4). Protein sequence coverage distribution showed that 39.5% of proteins fell within the 0–10% range, while molecular weights exhibited a broad distribution (>100 kDa). These results verify the reliability of the proteomic dataset for subsequent bioinformatics analysis.

Principal component analysis (PCA) was performed on the quantitative proteomic data from the wastewater-treated and control groups to assess replicate reproducibility. As shown in Figure S5, the centroids of the two groups exhibited distinct separation without overlap, indicating significant differences between the groups and high intra-group reproducibility. In addition, the cumulative distribution curves of the coefficient of variation (CV) for all identified proteins in both groups rose rapidly before leveling off (Figure S5). These results collectively demonstrate that exposure to tannery wastewater induced substantial changes in the protein expression profile of LL-1, while the overall reproducibility of the samples was robust. Therefore, the dataset is suitable for subsequent systematic functional annotation and pathway analysis.

### 3.4. Parameter-Free Transcriptomic Sequencing Analysis

**Figure 2:**
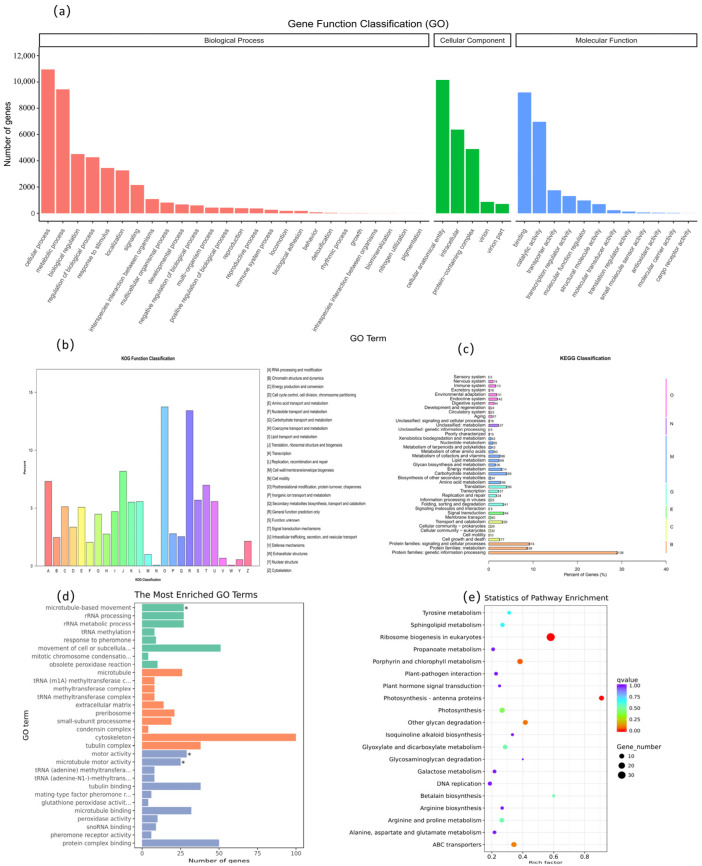
Pathway enrichment diagram of three databases in transcriptomics. (**a**) GO annotation classification statistical graph of LL-2 transcriptome. (*) GO pathway differential gene key expression (**b**) Statistical chart of KOG annotation classification in LL-2. (**c**) Classification and statistical diagram of KEGG metabolic pathways in LL-2. (**d**) Bar graph of GO pathway enrichment for differential genes between LL-2 and LL-3. (**e**) Scatter plot of KEGG pathway enrichment for differential genes between LL-2 and LL-3.

Functional annotation was performed on the de novo assembled transcriptome of strain LL-1 cultured under control conditions. Using the GO database, 17,193 unigenes were annotated and categorized into Biological Process, Cellular Component, and Molecular Function. Within these domains, the majority of unigenes were mapped to terms related to cellular processes, metabolic processes, cellular anatomical entities, binding, and catalytic activity (Figure 2a). For KOG annotation, 4023 unigenes were assigned to functional classes. The largest proportion was associated with post-translational modification, protein turnover, and chaperones, followed by translation, ribosomal structure and biogenesis, RNA processing, and signal transduction (Figure 2b). Furthermore, 3972 unigenes were mapped to the KEGG database. Topological analysis revealed that the most abundant functional clusters corresponded to genetic information processing, signaling and cellular processes, and metabolism (Figure 2c).

To elucidate the molecular response to stress, comparative transcriptomic analysis was performed between LL-1 cells cultured in tannery wastewater and the control group. A total of 4149 DEGs were identified, comprising 1508 upregulated and 2641 downregulated genes (|log_2_ Fold Change| > 1, q < 0.05; Figure S5). GO enrichment analysis revealed that under Biological Process, genes associated with “movement of cell or subcellular component” were most abundant, with “microtubule-based movement” identified as a significantly enriched term. For Cellular Component, genes related to the cytoskeleton and tubulin complex were highly represented. Similarly, under Molecular Function, enrichment was observed for protein complex binding, tubulin binding, and microtubule binding, alongside “motor activity” and “microtubule motor activity.” These findings suggest that the DEGs are closely associated with cell proliferation, morphogenesis, and intracellular transport, implying that LL-1 enhances the stability of its cytoplasmic microtubule system to maintain organelle and macromolecule function under stress (Figure 2d).

Furthermore, KEGG pathway analysis (q < 0.05) showed that DEGs were primarily enriched in ribosome biogenesis in eukaryotes, photosynthesis-antenna proteins, ABC transporters and porphyrin and chlorophyll metabolism. Notably, ribosome biogenesis in eukaryotes and photosynthesis-antenna proteins were the most significantly enriched pathways. Collectively, these results indicate that the DEGs are central to protein synthesis, redox homeostasis, photosynthesis, and pigment biosynthesis, underscoring the adaptive strategy of LL-1 to modulate these key metabolic pathways in response to wastewater stress (Figure 2e).

### 3.5. Label-Free Quantitative Proteomics Analysis

**Figure 3:**
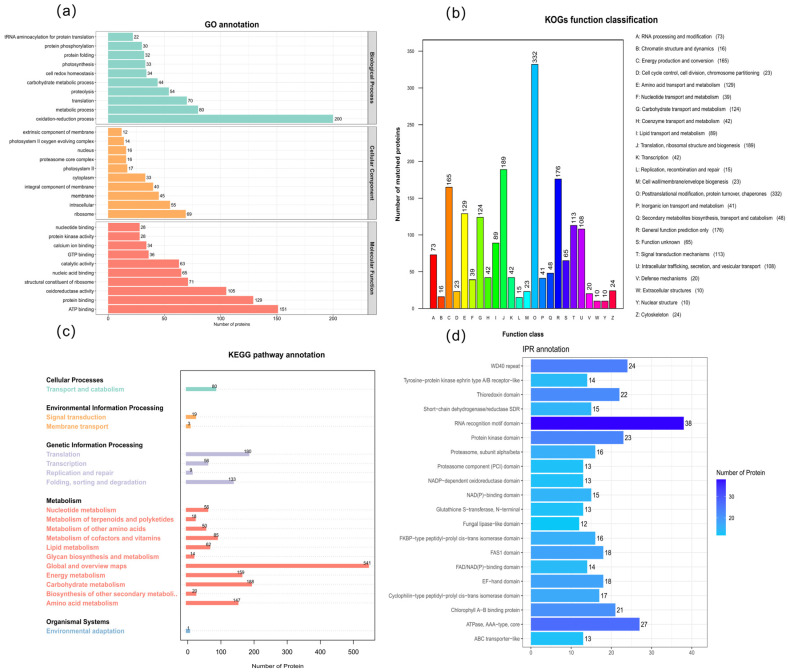
Pathway enrichment status of LL-2 proteomics across various databases. (**a**) Bar graph of GO annotation results for LL-2 proteomics (**b**) Bar graph of LL-2 proteomics KOG annotation results (**c**) Bar graph of KEGG annotation results for LL-2 proteomics. (**d**) Bar graph of LL-2 proteomic domain annotation results.

Functional annotation of the control group proteome was performed using the GO, KEGG, and KOG databases. GO analysis revealed that under Biological Process, proteins involved in the oxidation–reduction process, metabolic process, and translation were the most abundant. For Cellular Component, proteins were primarily located in the ribosome, intracellular regions, and membranes. In the Molecular Function category, ATP binding predominated, followed by oxidoreductase activity and protein binding (Figure 3a). KOG annotation indicated that the largest functional group was post-translational modification, protein turnover, and chaperones. Other major categories included translation, ribosomal structure and biogenesis and energy production and conversion (Figure 3b). KEGG analysis classified the proteins into five major functional clusters, Global and overview maps encompassed the highest number of proteins, followed by carbohydrate metabolism and translation (Figure 3c). Furthermore, InterProScan analysis of functionally uncharacterized proteins identified 20 InterPro (IPR) domains. The RNA recognition motif was the most frequent, followed by the ATPase AAA-type core, WD40 repeat, protein kinase, and thioredoxin domains (Figure 3d).

**Figure 4:**
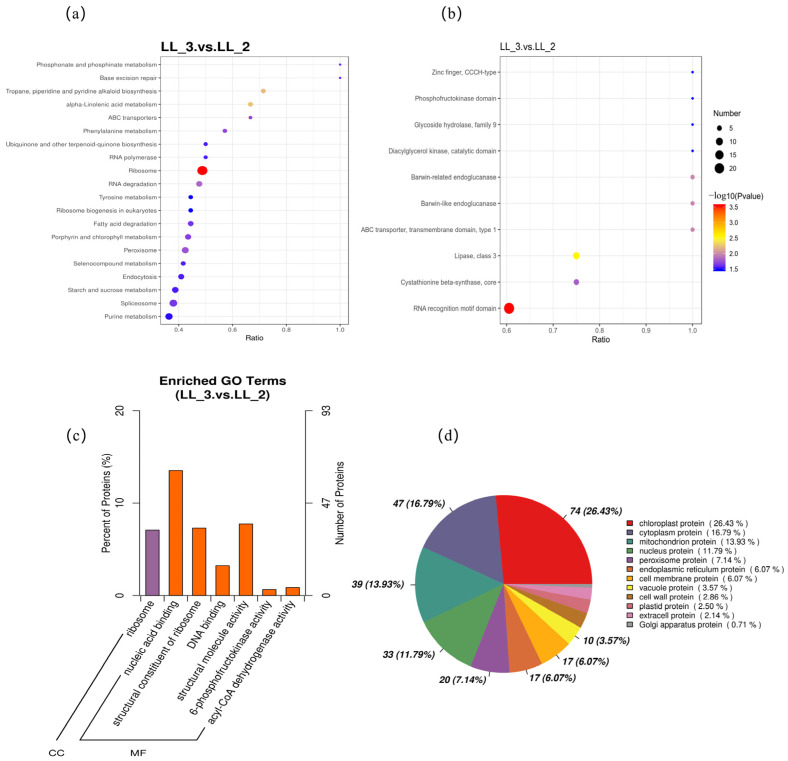
Pathway annotation of LL-3 differential proteins in various databases. (**a**) LL-3 differential protein KEGG enrichment bubble chart. (**b**) Bubble plot of enriched structural domains of LL-3 differential proteins. (**c**) GO enrichment bar chart of LL-3 differential proteins. (**d**) Subcellular localization analysis of LL-3 differential proteins.

Differential expression analysis was performed using the control group as a reference to characterize the proteomic response to tannery wastewater stress. Functional enrichment analysis was subsequently conducted to elucidate the key adaptive pathways of LL-1. A total of 2761 proteins were identified, of which 1052 were differentially expressed (409 upregulated and 643 downregulated; FC > 2.0 or <0.5; Figure S5). GO enrichment analysis revealed that DEPs were predominantly associated with terms including nucleic acid binding, structural constituent of ribosome, DNA binding, structural molecule activity, 6-phosphofructokinase activity, and acyl-CoA dehydrogenase activity. These results suggest that tannery wastewater stress stimulates physiological activities related to nucleic acid metabolism and enzymatic regulation in LL-1 (Figure 4c). Furthermore, KEGG pathway analysis indicated significant enrichment in phosphonate and phosphinate metabolism, base excision repair, piperidine and pyridine alkaloid biosynthesis, alpha-linolenic acid metabolism, and ABC transporters. These findings imply that LL-1 adapts to pollution stress by modulating metabolite biosynthesis and membrane selective permeability (Figure 4a).

Protein domain enrichment analysis offered deeper insights into protein function. Highly enriched domains included zinc finger CCCH-type, phosphofructokinase domain, glycoside hydrolase family 9, diacylglycerol kinase catalytic domain, and Barwin-related/like endoglucanases. These results indicate that LL-1 enhances stress tolerance by activating key enzymes involved in carbohydrate and lipid metabolism and by regulating defense-related proteins (Figure 4b). Subcellular localization analysis further revealed that DEPs were primarily distributed in the chloroplast (26.43%), cytoplasm (16.79%), mitochondrion (13.93%), and nucleus (11.79%), suggesting that these organelles serve as the primary sites for metabolic regulation and stress response (Figure 4d).

## 4. Discussion

Transcriptomic profiling revealed that the cytoplasmic microtubule system of LL-1 exhibited the most pronounced upregulation of DEGs under tannery wastewater stress. This suggests a compensatory remodeling mechanism, as microtubules are critical for intracellular transport, spindle assembly, and mitosis; their reorganization is likely essential to sustain cell division and nutrient transport under pollutant stress [30,31]. Given that microtubules are integral to the cytoskeletal network, their stability directly determines the efficiency of cellular transport and division. The concurrent upregulation of cytoskeleton-related genes was likely triggered by the intracellular accumulation of ammonium ions, activating protective pathways to ensure cell survival [32]. These findings align with stress responses observed in other microalgae. For instance, in *Scenedesmus* sp. BHU1 exposed to high salinity, the upregulation of cytoskeleton and microtubule-associated proteins was reported to maintain cellular integrity amid osmotic stress [33]. Similarly, Chlorella vulgaris exhibited enhanced expression of tubulin and motor activity genes under heavy metal or ammonia stress, pointing to a conserved adaptive mechanism involving the stabilization of cytoplasmic structures to facilitate intracellular transport during pollutant exposure [34].

KEGG pathway analysis revealed a significant upregulation of genes involved in ribosome biogenesis in eukaryotes and photosynthesis-antenna proteins. Ribosome biogenesis is an energy-intensive process requiring the coordination of three RNA polymerases; consequently, it is prioritized during cellular resource redistribution under stress [35]. Previous studies have established that wastewater exposure typically induces oxidative stress and necessitates protein homeostasis reprogramming, often manifested as the upregulation of ribosome-related genes. This transcriptional response enhances translational and repair capacities [36,37], a pattern consistent with the observations in LL-1. These findings suggest that under tannery wastewater stress, LL-1 upregulates protein synthesis to restore translational capacity and replace damaged proteins, thereby sustaining essential cellular functions. This mechanism mirrors findings in other microalgae, for instance, Chlorella vulgaris exposed to high-ammonia swine wastewater exhibited upregulated ribosome biogenesis to meet translational demands, coupled with adjustments in light-harvesting proteins to mitigate oxidative damage. Furthermore, the upregulation of ABC transporters—observed here and reported in Chlorella and Scenedesmus under heavy metal stress—facilitates toxicant efflux and homeostasis maintenance. Collectively, these parallels indicate that LL-1 employs broadly conserved molecular adaptations, characterized by enhanced protein turnover, optimized light harvesting, and active transport, to cope with the multifaceted stressors in tannery wastewater.

At the proteomic level, GO enrichment analysis revealed significant enrichment of DEPs in categories such as nucleic acid binding and ribosomal structural constituents. This response is likely triggered by high concentrations of ammonium and other pollutants in tannery wastewater, which induce oxidative stress and nutrient imbalances. To facilitate adaptation, the microalga must rapidly modulate gene expression, a process necessitating the extensive involvement of nucleic acid-binding proteins [38]. Furthermore, as the primary site of protein synthesis, the ribosome plays a critical role in translation, and its regulation is intrinsically linked to the observed proteomic shifts [39]. Notably, functional enrichment highlighted DEPs associated with 6-PFK and ACAD activity, pointing to energy metabolism reprogramming. The upregulation of 6-PFK, a rate-limiting enzyme in glycolysis, suggests that LL-1 accelerates glycolytic flux to boost ATP production and carbon flow towards acetyl-CoA [40]. Concurrently, the enrichment of ACAD activity indicates enhanced lipid mobilization via fatty acid β-oxidation, thereby augmenting energy supply and potentially aiding in pollutant degradation [41]. KEGG pathway analysis further implicated alkaloid biosynthesis and α-linolenic acid metabolism in the stress response (*p* < 0.05). However, it is important to note that these insights are derived from transcriptomic and proteomic data; targeted metabolomic validation is required to confirm actual fluctuations in metabolite levels and pathway fluxes [42]. Synthesizing these findings, it can be inferred that LL-1 copes with wastewater stress by upregulating efflux mechanisms to expel toxic by-products and by initiating DNA repair mechanisms to maintain genomic stability [43].

While the observed responses—such as the upregulation of ribosome biogenesis and light-harvesting proteins—align with reports in other microalgae exposed to wastewater or heavy metals, stress responses may exhibit species-specific variations. For instance, some species prioritize photoprotective repair mechanisms under comparable stressors, whereas others favor energy reallocation strategies. Although the patterns identified herein suggest that LL-1 employs broadly conserved adaptive mechanisms, potential species-specific nuances remain to be elucidated through comparative genomic or transcriptomic analyses.

It is also important to acknowledge that the physicochemical complexity of tannery wastewater (e.g., pH fluctuations, dissolved oxygen levels, and trace metals) may introduce confounding factors affecting expression profiles. Specifically, low dissolved oxygen can induce anaerobic metabolic genes, while heavy metals (e.g., chromium) activate specific detoxification pathways. Furthermore, pH shifts can modulate free ammonia toxicity and membrane permeability. These variables likely exert synergistic effects on ribosome biogenesis, photosynthetic adjustment, and cytoskeleton remodeling, thereby shaping the overall stress response [44]. Given the focus on real-world wastewater treatment, this study did not isolate these physicochemical variables. Consequently, distinguishing their individual contributions to the observed molecular phenotypes remains challenging. Future investigations will address this limitation using controlled, factorial-design experiments to systematically decouple the effects of individual and combined environmental factors.

To elucidate the molecular mechanisms governing the algal response to tannery wastewater, a conjoint transcriptomic and proteomic analysis was performed. This integration identified 243 concordant targets, with 56 concordantly upregulated and 45 concordantly downregulated gene-protein pairs, indicating extensive genetic and proteomic reprogramming (Figure S6). GO enrichment analysis revealed that these shared targets were primarily associated with macromolecular and primary metabolic processes, specifically nucleic acid binding, ribosomal structural components, and metal-ion binding. These results imply that LL-1 prioritizes ribosome assembly and the regulation of nucleic acid-binding proteins to preserve translational efficiency and metabolic homeostasis [45]. This observation aligns with Wang et al. [46], who reported that microalgae exposed to complex wastewater matrices activate ribosomal translation to establish a structural and energetic foundation for adaptation. Regarding Cellular Components, enrichment was concentrated in membrane composition and transport functions. This suggests that LL-1 mitigates chemical stress by modulating transmembrane proteins and transport systems. Previous studies have demonstrated that algae upregulate ABC and MFS transporters under heavy metal or ammonia stress to facilitate pollutant efflux [47]. The present study corroborates this mechanism, highlighting active transport as a critical barrier against toxic stress [48] (Figure S7). Furthermore, integrated KEGG pathway analysis showed that DEGs and DEPs were co-enriched in critical pathways, including starch and sucrose metabolism, porphyrin and chlorophyll metabolism, and aminoacyl-tRNA biosynthesis. The activation of these pathways suggests that LL-1 bolsters photosynthesis and carbon fixation to augment ATP and reducing power supply, thereby supporting energy-intensive repair mechanisms [49]. Analogous findings were reported in Scenedesmus under wastewater stress, where metabolic remodeling of carbohydrates and lipids provided rapid energy for adaptation [50] (Figure S8).

## 5. Conclusions

This study investigated the growth and ammonia nitrogen removal of a strain of green microalga isolated from the Lalu Wetland on the Tibetan Plateau in tannery wastewater. Based on multi-omics analysis, the following conclusions were drawn: The strain was identified as a member of the genus Micractinium and designated as *Micractinium* sp. LL-1. Following inoculation into tannery wastewater, the ammonia nitrogen concentration decreased rapidly, achieving a removal efficiency of 98.7%. The maximum accumulated biomass reached 1641.7 mg/L in BG11 medium and 1461.3 mg/L in wastewater. Under tannery wastewater stress, transcriptomic analysis identified 4353 DEGs involved in cell proliferation, morphogenesis, intracellular transport, protein synthesis, photosynthesis, and redox processes. These DEGs were significantly enriched in ribosome biogenesis and light-harvesting antenna protein pathways and were associated with enhanced microtubule stability, which helps maintain organelle function. Proteomic analysis identified 1052 DEPs. The upregulation of ATP-binding proteins, oxidoreductases, and acyl-CoA dehydrogenases activated glycolysis and fatty-acid β-oxidation, thereby restructuring cellular energy metabolism. Concurrently, the increased expression of ABC transporters and base excision repair proteins enhanced pollutant efflux and DNA-damage repair capacity. Integrated transcriptomic and proteomic analyses indicated that the strain balances energy metabolism through photosynthesis and carbon fixation while coordinating nitrogen, glucose, and phosphate transport pathways; it also synthesizes nitrogen-containing secondary metabolites, such as piperidine and pyridine alkaloids, in response to stress. Subcellular localization analysis indicated that the chloroplast (26.4%), cytoplasm (16.8%), and mitochondrion (13.9%) are the primary sites of the stress response. Overall, this study expands the microbial resource database of Tibetan Plateau wetlands, provides insights into the taxonomy and environmental adaptation mechanisms of Micractinium, and offers a theoretical foundation and a potential algal candidate for the bioremediation and resource recovery of industrial wastewater.

## Data Availability

The original contributions presented in this study are included in the article. Further inquiries can be directed to the corresponding authors.

## Supplementary Materials

The following supporting information can be downloaded at: https://www.mdpi.com/article/10.3390/biology15020123/s1, Table S1: Physicochemical properties of wastewater; Table S2: Ten 18S rRNA sequences closely related to *Micractinium* sp. LL-1; Table S3: Summary of sample sequencing data quality; Table S4: Distribution of transcript splicing length; Figure S1: NJ phylogenetic tree constructed based on 18S r RNA; Figure S2: Distribution of sequencing data error rates; Figure S3: GC content distribution; Figure S4: (a) Sample protein identification overview map; (b) Peptide length range distribution; (c) Parent ion mass tolerance profile; (d) Map of the number distribution of unique peptides in the identified protein; (e) Protein coverage distribution map; (f) Protein molecular weight distribution map; Figure S5: (a) LL_3 vs LL_2 Differential gene volcano map. (b) Differential protein volcano map. (c) PCA analysis diagram. (d) Repeatability CV analysis chart. Figure S6: Correlation analysis of transcriptome and proteome expression; Figure S7: GO function rich cluster heat map; Figure S8: Heat map of KEGG function rich cluster.
