# Supplementary material for: Integrated Transcriptomic and Proteomic Analysis of the Stress Response Mechanisms of Micractinium from the Tibetan Plateau Under Leather Wastewater Exposure
Source: Biology (Basel). 2026 Jan 9;15(2):123. doi: 10.3390/biology15020123 (PMC12837426; doi:10.3390/biology15020123)
Supplement: Supplementary file 1 [file biology-15-00123-s001.zip › biology-4017860-supplementary.pdf]

1. Physicochemical properties of leather wastewater

The tanning wastewater used in this study was collected from the effluent outlet of a leather factory in Lhasa, which had been discharged after undergoing multiple treatments. The leather industry of this factory belongs to a distinctive and high-quality ecological industry, paying more attention to green ecology and green economy compared to the traditional leather industry. After wastewater collection, a multi-functional water quality tester was immediately used to measure the pH value, total dissolved solids, conductivity, and other indicators of the water sample.

Table S1. Physicochemical properties of wastewater

Indicator	Wastewater
Temperature	18.1±0.9℃
pH	6.96±0.24
EC	671±16 μs
TDS	474±12 ppm
SALT	383±7 ppm
Turbidity	3.23±0.15 NTU

2. Obtain microalgae identification

To accurately identify the species of algal strain LL-1, the 18S rRNA method, which is widely used in the field of microalgal taxonomy, was employed for molecular biological identification. The gene sequence obtained from sequencing was aligned with relevant sequences in the GenBank database to identify species closely related to the algal strain and obtain the sequences of these species. Subsequently, a phylogenetic tree was constructed through sequence searching, selection, alignment, tree construction, and modification, and the species of the algal strain was determined by combining the NCBI (National Center for Biotechnology Information) database and Eztaxon mode.

Table S2. Ten 18S rRNA sequences closely related to *Micractinium sp.* LL-1.

Description	Scientific Name	Max Score	Total Score	Query Cover	Per. Ident	Accession
Micractinium sp. CCAP 248/13 18S rRNA gene (partial), ITS1, 5.8S rRNA gene, ITS2 and 28S rRNA gene (partial), strain CCAP 248/13	Micractinium sp.CCAP 248/13	695	695	100%	98.23%	FM205865.1
Micractinium pusillum 18S ribosomal RNA gene, partial sequence	Micractinium pusillum	695	695	100%	98.23%	AF237662.1
Micractinium sp. MM0001 small subunit ribosomal RNA gene, partial sequence	Micractinium sp. MM0001	689	689	100%	97.98%	MF959935.1

Micractinium pusillum isolate Zhalong Salt Lake 1 small subunit ribosomal RNA gene, partial sequence	Micractinium pusillum	689	689	100%	97.98%	MK764917.1
Chlorella sp. YACCYB105 18S ribosomal RNA gene, partial sequence	Chlorella sp. YACCYB105	689	689	100%	97.98%	MH619552.1
Chlorella sp. YACCYB104 18S ribosomal RNA gene, partial sequence	Chlorella sp. YACCYB104	689	689	100%	97.98%	MH619551.1
Chlorella sp. YACCYB103 18S ribosomal RNA gene, partial sequence	Chlorella sp. YACCYB103	689	689	100%	97.98%	MH619550.1
Chlorella sp. YACCYB102 18S ribosomal RNA gene, partial sequence	Chlorella sp. YACCYB102	689	689	100%	97.98%	MH619549.1
Chlorella sp. YACCYB101 18S ribosomal RNA gene, partial sequence	Chlorella sp. YACCYB101	689	689	100%	97.98%	MH619548.1
Chlorella sp. YACCYB100 18S ribosomal RNA gene, partial sequence	Chlorella sp. YACCYB100	689	689	100%	97.98%	MH619547.1

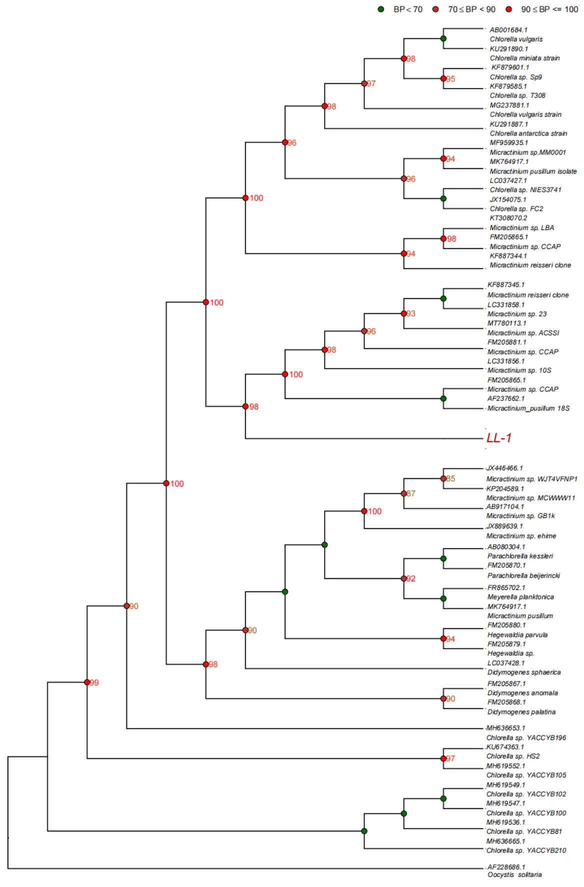

Figure S1 NJ phylogenetic tree constructed based on 18S r RNA

3. Sample RNA quality detection and sequencing data quality control

Total RNA was extracted from two sets of six algal cell samples, and the integrity and total amount of the sample total RNA were tested. Subsequently, the distribution of sequencing error rates was examined, as this indicator can reflect the quality of sequencing data to a certain extent. Finally, the NEB common library construction method was used to examine the distribution of A/T/G/C content. The Q30 base percentage was above 94.84%, indicating that the transcript sequencing data quality was good. The N50 values were 2934 and 2586, respectively. The longer the Unigene N50, the fewer the number, indicating better assembly quality.

Table S3. Summary of sample sequencing data quality

sample	raw_reads （bp）	clean_reads （bp）	error_rate （%）	Q20 （%）	Q30 （%）	GC_pct （%）
LL_2_1	22846006	21931235	0.02	98.19	94.95	58.40
LL_2_2	23003577	22110794	0.02	98.16	94.84	58.38
LL_2_3	23596432	22213109	0.02	98.28	95.20	58.46
LL_3_1	23115526	22306823	0.02	98.17	94.86	58.28
LL_3_2	26266179	24476184	0.02	98.26	95.06	57.99
LL_3_3	23340666	21968313	0.02	98.25	95.10	57.80

Table S4. Distribution of transcript splicing length

Type	Min length （bp）	Mean length （bp）	Median length （bp）	Max length （bp）	N50	N90	Total nucleotides
Transcript	301	1930	1410	21286	2934	928	130829762
Unigene	301	1613	1050	21286	2586	669	50134160

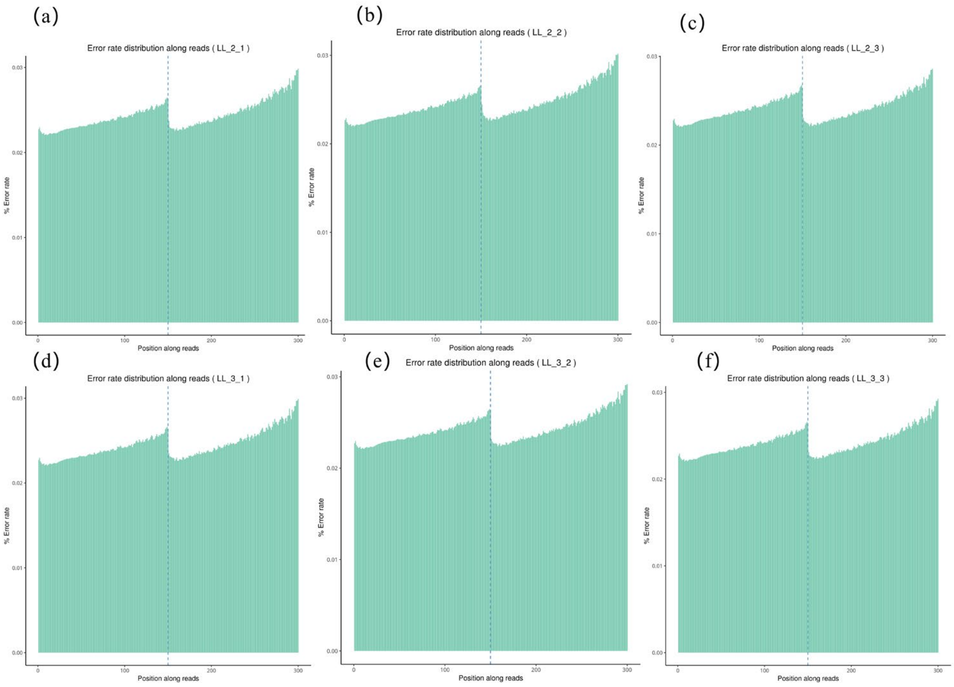

Figure S2 Distribution of sequencing data error rates(a) LL_2_1; (b) LL_2_3; (c) LL_3_1; (e) LL_3_2; (f) LL_3_3

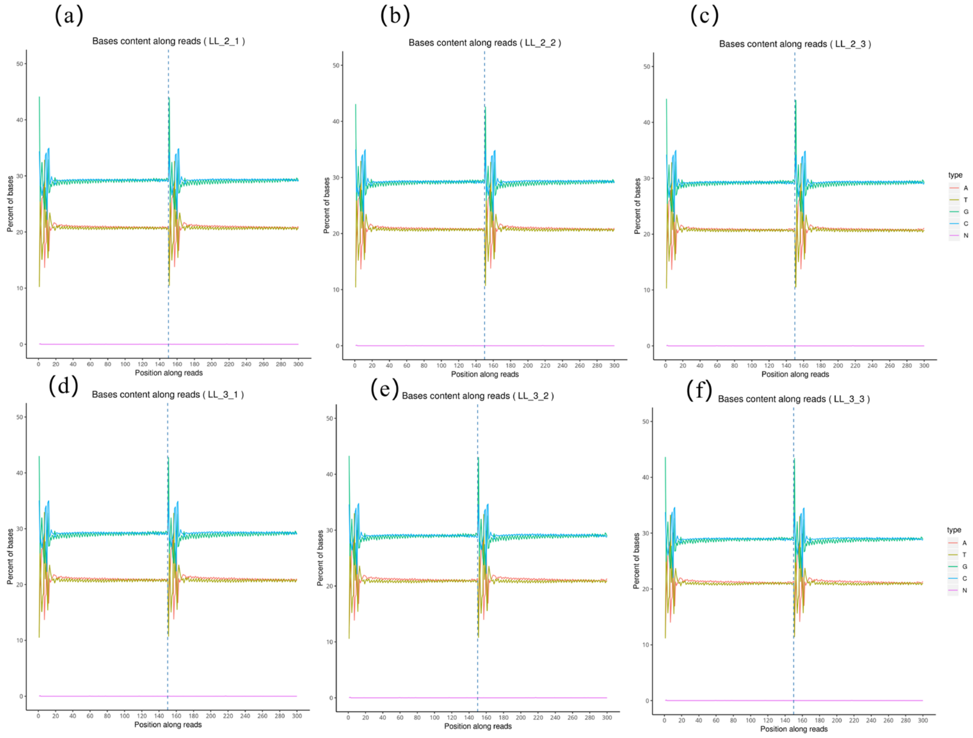

Figure S3 GC content distribution (a) LL_2_1; (b) LL_2_3; (c) LL_3_1; (e) LL_3_2; (f) LL_3_3

4. Sample protein quality detection and data quality control

The Proteome Discoverer software was used to filter and search for reliable peptide spectra (PSMs) and reliable proteins for FDR verification. Peptides and proteins with an FDR < 1% were retained, resulting in a final number of 19,093 peptides and 3,106 proteins. The peptide lengths were mainly distributed between 7 and 23, which is within the standard peptide length range. The mass deviation between the measured molecular weight and theoretical molecular weight of the peptide precursor ions was relatively small. Protein coverage and protein molecular weight distribution were examined. The sample contained the highest number of proteins within the 0-0.1 coverage range, accounting for 39.5%. The molecular weight of proteins could reach up to over 100 Daltons, with a wide distribution range, indicating a broad range of identified proteins.

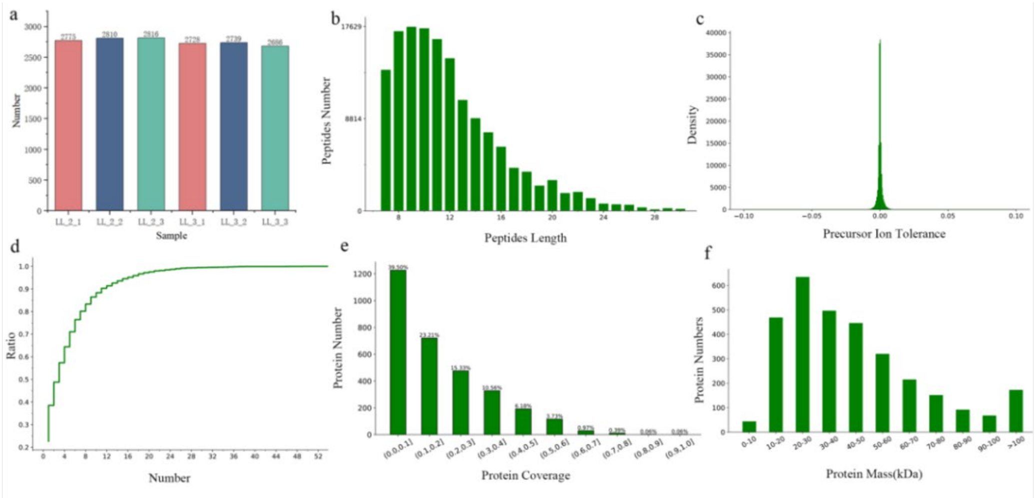

Figure S4 (a) Sample protein identification overview map;(b) Peptide length range distribution; (c) Parent ion mass tolerance profile; (d) Map of the number distribution of unique peptides in the identified protein; (e) Protein coverage distribution map; (f) Protein molecular weight distribution map

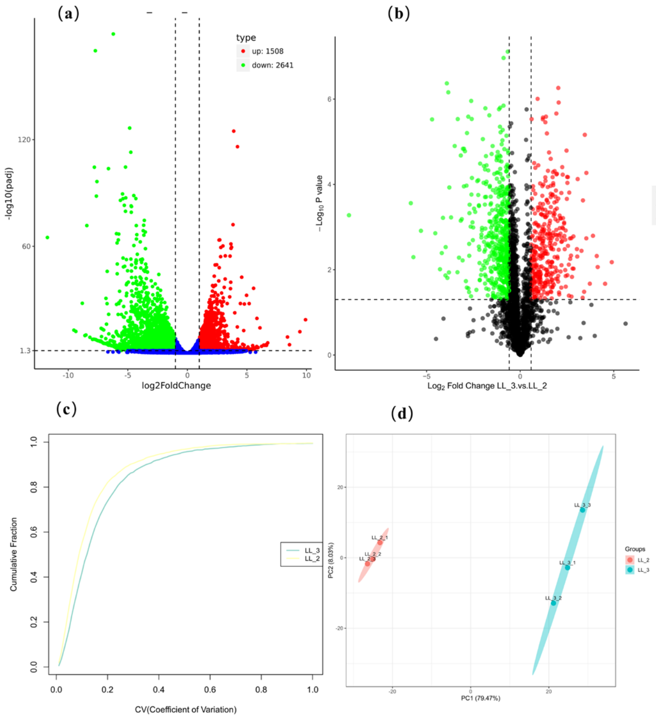

Figure S5 (a) LL_3 vs LL_2 Differential gene volcano map. (b) Differential protein volcano map. (c) PCA analysis diagram. (d) Repeatability CV analysis chart.

Appendix A.4 Expression level correlation analysis

To deeply explore the potential relationship between gene and protein expression levels and reveal the interactions between different omics levels, utilized the Pearson correlation coefficient to analyze the correlation between the differential fold changes of genes jointly identified in the transcriptome and proteome across the two omics.

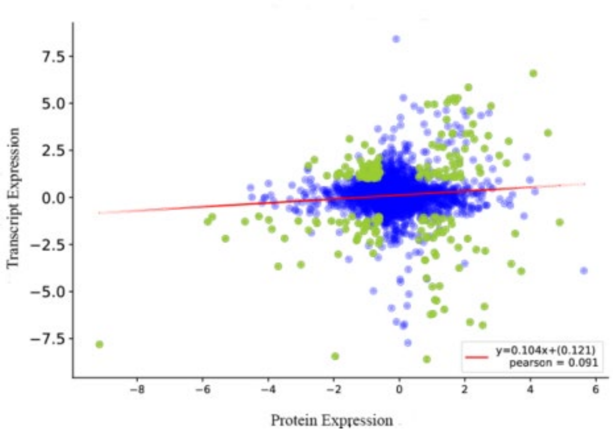

Figure S6 Correlation analysis of transcriptome and proteome expression

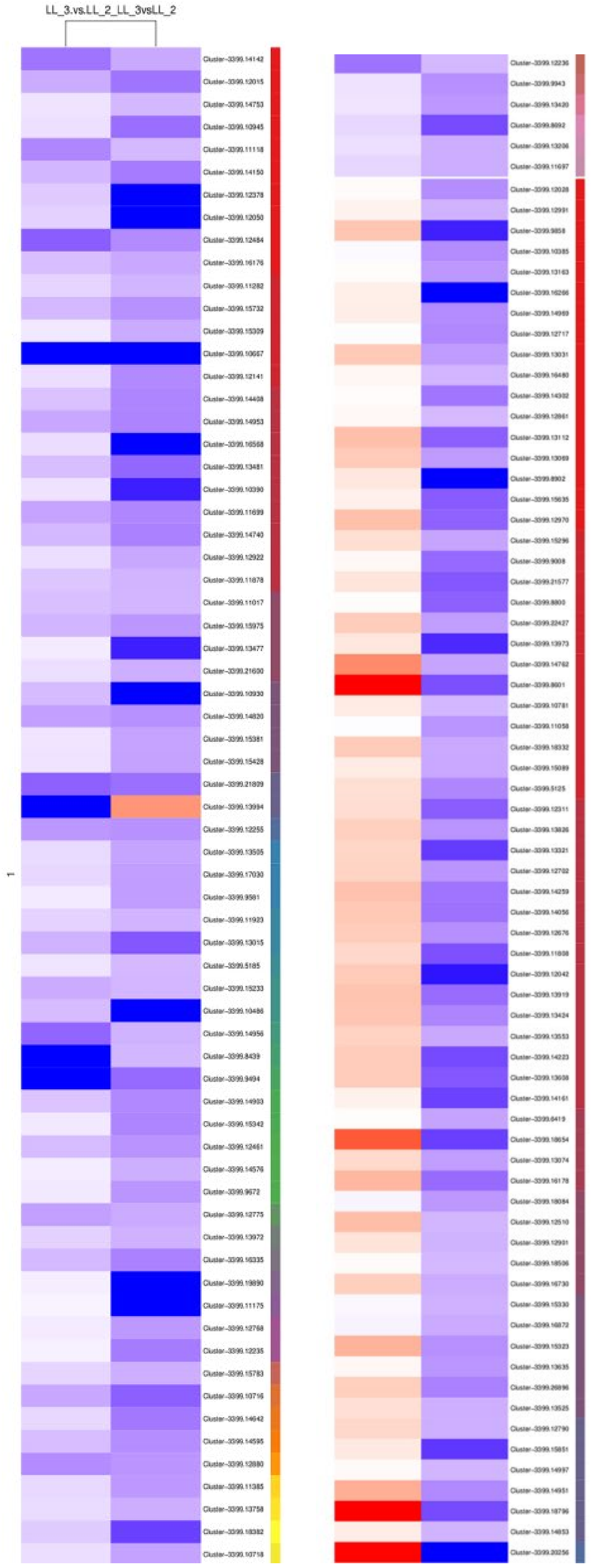
LL_3.vs.LL_2_LL_3vsLL_2

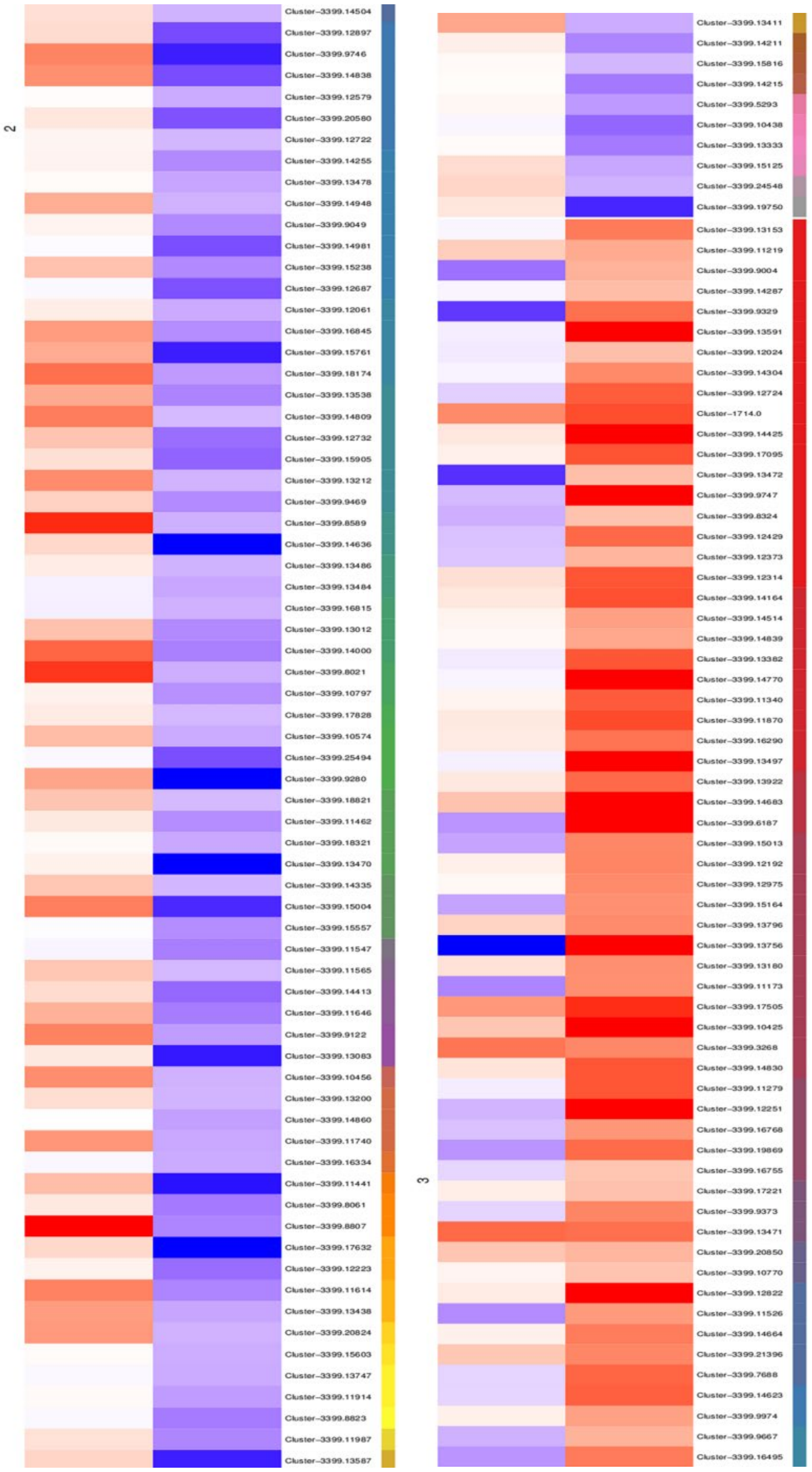

Figure S7. GO function rich cluster heat map

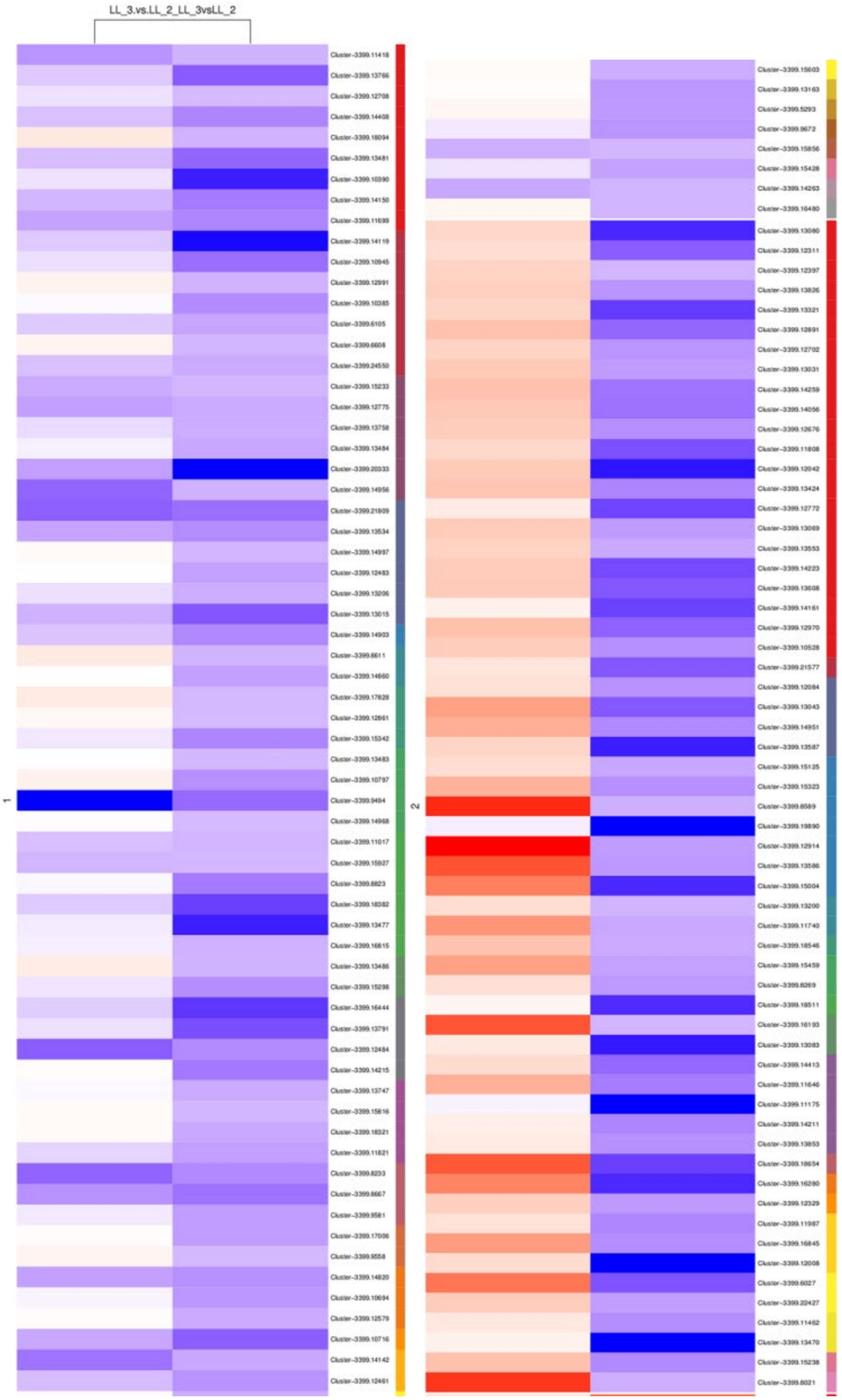

Figure S8 Heat map of KEGG function rich cluster
